# Case Report: Curative effect of OA-PICA protected bypass in severe PICA-complicated vertebral artery stenosis treatment: Results in 3 cases

**DOI:** 10.3389/fsurg.2023.1074438

**Published:** 2023-02-13

**Authors:** Liming Zhao, Bingqian Xue, Gaochao Guo, Ruiyu Wu, Tao Gao, Yang Liu, Yuxue Sun, Juha Hernesniemi, Hugo Andrade Barazarte, Tianxiao Li, Chaoyue Li

**Affiliations:** ^1^ Department of Neurosurgery, Zhengzhou University People's Hospital, Henan Provincial People's Hospital, Zhengzhou, China; ^2^ Department of Neurosurgery, Henan University People's Hospital, Henan Provincial People's Hospital, Zhengzhou, China

**Keywords:** occipital artery (OA), posterior internal carotid artery, artery stenosis, protective bypass, treatment

## Abstract

**Objectives:**

We aimed to explore the results of OA-PICA-protected bypass grafting in patients with severe stenosis of the vertebral artery combined with PICA.

**Methods:**

Three patients with vertebral artery stenosis involving the posterior inferior cerebellar artery, treated by the Department of Neurosurgery of Henan Provincial People's Hospital from January 2018 to December 2021, were retrospectively analyzed. All the patients underwent Occipital Artery–Posterior Inferior Cerebellar Artery (OA-PICA) bypass surgery followed by elective vertebral artery stenting. Intraoperative indocyanine green fluorescence angiography (ICGA) showed patency of the bridge-vessel anastomosis. Postoperatively, the ANSYS software was used to assess the flow pressure changes and vascular shear in combination with the reviewed DSA angiogram. CTA or DSA was reviewed 1–2 years postoperatively, and the prognosis was evaluated by the modified Rankin Scale (mRS) one year postoperatively.

**Results:**

OA-PICA bypass surgery was completed in all patients, with intraoperative ICGA showing a patent bridge anastomosis, followed by stenting of the vertebral artery, and a review of the DSA angiogram. We also employed ANSYS software evaluation of the bypass vessel, which showed stable pressure and low turnover angle, suggesting a low rate of long-term occlusion of the vessel. All patients had no procedure-related complications during their hospitalization, and were followed up for a mean of 24 months postoperatively, with a good prognosis (mRS score of 1) at 1 year postoperatively.

**Conclusion:**

OA-PICA-protected bypass grafting is an effective treatment for patients with severe stenosis of the vertebral artery combined with PICA.

## Introduction

With endovascular therapy advances, vertebral artery angioplasty has become an important treatment for arterial stenosis. However, patients with vertebral artery stenosis, stent placement or balloon dilation may induce thrombosis in the diseased vessel, causing the unstable plaque in the diseased vessel to dislodge and block the opening of one or more penetrating arteries, leading to postoperative cerebral infarction. The posterior inferior cerebellar artery (PICA) is the largest branch of the vertebral artery, located in the skull. Its anterior medullary, lateral medullary, and medullary segments of the tonsil are divided into important branches that supply blood to the brainstem. When a plaque of vertebral artery stenosis is in the posterior inferior cerebellar artery (PICA), the risk of stent angioplasty is higher, in the early VA stage of severe PICA stenosis, only stents were placed, and some patients experienced postoperative infarction caused by PICA occlusion. Therefore, based on skilled surgical techniques, we proposed a protected bypass for posterior circulation PICA segment stenosis.

## Materials and methods

### General information

A total number of three patients with vertebral artery stenosis involving the posterior inferior cerebellar artery who were hospitalized in the Department of Neurosurgery of Henan Provincial People's Hospital from January 2018 to December 2021 were selected. All were male, aged 48–59 years (mean age of 52.67 years). They mainly presented with dizziness, accompanied by headache in two cases and blurred vision in one case. The values of the modified Rankin Scale (mRS) scores were within 1–2 at admission, with an average of 1.67. All patients underwent OA-PICA-protected bypass surgery. The clinical data of the patients are presented in Table 1.

**Table 1:** Clinical profile of the patients with severe vertebral artery stenosis involving the posterior inferior cerebellar artery.

Csae.	Gender	Age (Years)	Clinical symptoms	Surgery method	Time between surgeries	mRS	
						Pre-surgery	Post-surgery
1	Male	48	Headache and dizziness	OA-PICA-protected bypass + elective vertebral artery stenting	6d	1	1
2	Male	59	Headache and dizziness	OA-PICA-protected bypass + elective vertebral artery stenting	10d	2	1
3	Male	53	Dizziness with blurred vision	OA-PICA-protected bypass + elective vertebral artery stenting	7d	2	1

### Preoperative evaluation

All patients underwent preoperative DSA to assess the development of bilateral vertebral arteries, the degree of stenosis of the affected vertebral artery, and the involvement of the posterior inferior cerebellar artery. Preoperative magnetic resonance perfusion PWI was performed to determine the ischemia-induced changes of the posterior circulation. We also conducted an assessment of the diameter and the course of the occipital artery, and did a preoperative ultrasound examination of the scalp for OA tracing.

### Surgical procedure

**Figure 1:**
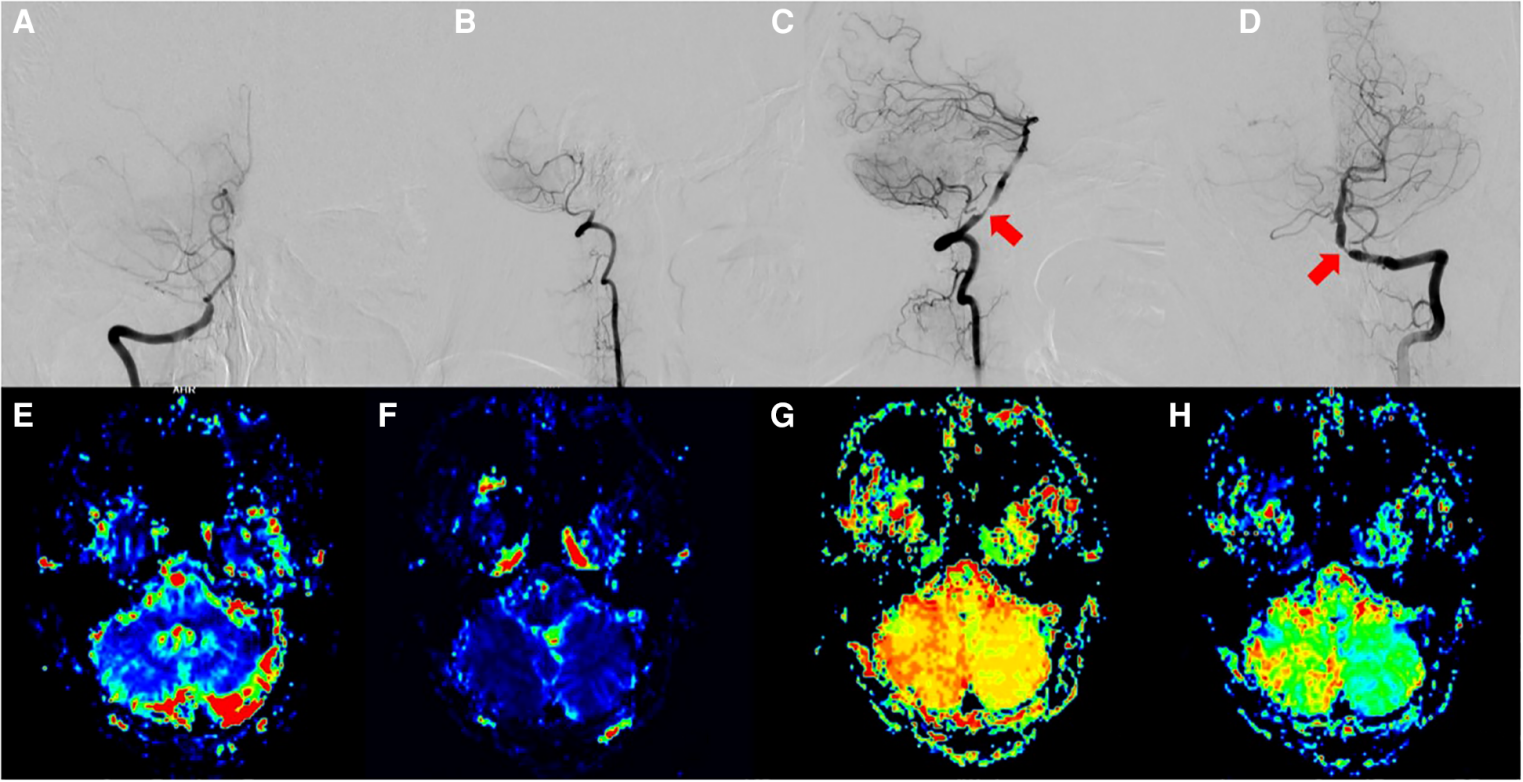
(**A,B**) Right vertebral artery occlusion; (**C,D**) Left dominant vertebral artery with stenosis at the opening of the vertebral and PICA arteries; (**E–H**) MRI, CBV, CBF, TTP, and MTT results indicate bilaterally reduced perfusion and intracranial ischemia.

The patient was placed in the lateral prone position, and a surgical incision was made from the C4 level along the midline up to the external occipital ridge, parallel to the superior nuchal line outward to the posterior border of the mastoid process. Then, the occipital artery was dissected and freed under a surgical microscope and flushed with heparinized saline. Indocyanine green fluorescence angiography (ICGA) was next performed to observe the patency of the bridge-vessel anastomosis (Figure 1), followed by elective vertebral artery stenting.

### Prognostic evaluation

We recorded the surgery-related complications during hospitalization; Postoperatively, ANSYS software was applied in combination with DSA angiogram reviews to assess the changes in the blood flow pressure and the vascular shear. We also evaluated the time and frequency of the postoperative review. Postoperatively, CTA or DSA was applied to assess the patency of the bridge-vessel anastomosis, whereas PWI was implemented to determine the improvement of ischemia in the patients. From 6 to 12 months postoperatively, the improvement of the clinical symptoms and the prognosis of the patients were evaluated using the mRS scale. mRS ≤ 2 was considered to indicate good prognosis, whereas >2 represented poor prognosis.

## Results

In the three patients selected, DSA confirmed the dominance of the affected vertebral artery. Severe occlusion of the contralateral vertebral artery was observed in two cases (case 1 and case 3), and congenital absence of the contralateral vertebral artery was detected in one case (case 2). In case 2, the affected vertebral artery had ≥80% stenosis at the beginning of the PICA, whereas had ≥90% stenosis was established in case 1 and case 3. The occipital artery diameter at the superior collateral line was within 1.20–1.50 mm, with a mean of 1.35 mm (Figure 1).

**Figure 2:**
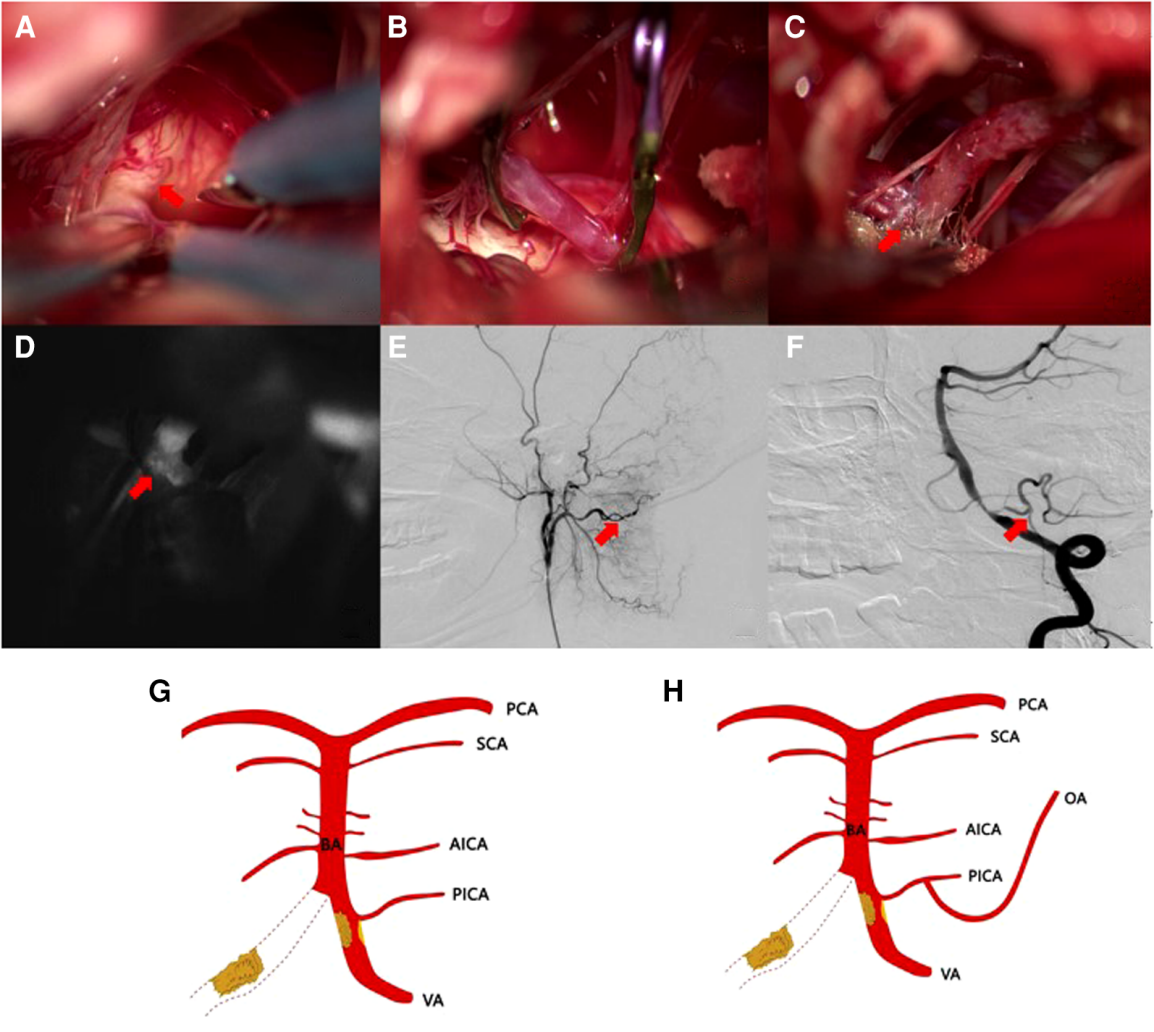
(**A**) Severe calcification of the vertebral artery and the PICA opening; (**B**) OA-PICA bypass at the caudal loop of the PICA artery; (**C,D**) Post-vascular anastomosis patent vessel displayed by fluoroscopy; (**E,F**) A grafted vessel (arrow) in a postoperative angiography image. (**G,H**) Schematic drawing of the OA-PICA bypass.

OA-PICA-protected bypass grafting was completed in all three patients, and intraoperative ICGA showed patent bridge-vessel anastomosis. One patient (case 2) underwent vertebral artery stenting on postoperative day 10 because of blood leakage in the operative area; the stenting of the remaining two cases was completed within seven days postoperatively. Intraoperative angiography showed that all the bypass vessels were patent and well supplied with blood (Figures 2). None of the patients had neurological function impairment-related complications during hospitalization.

**Figure 3:**
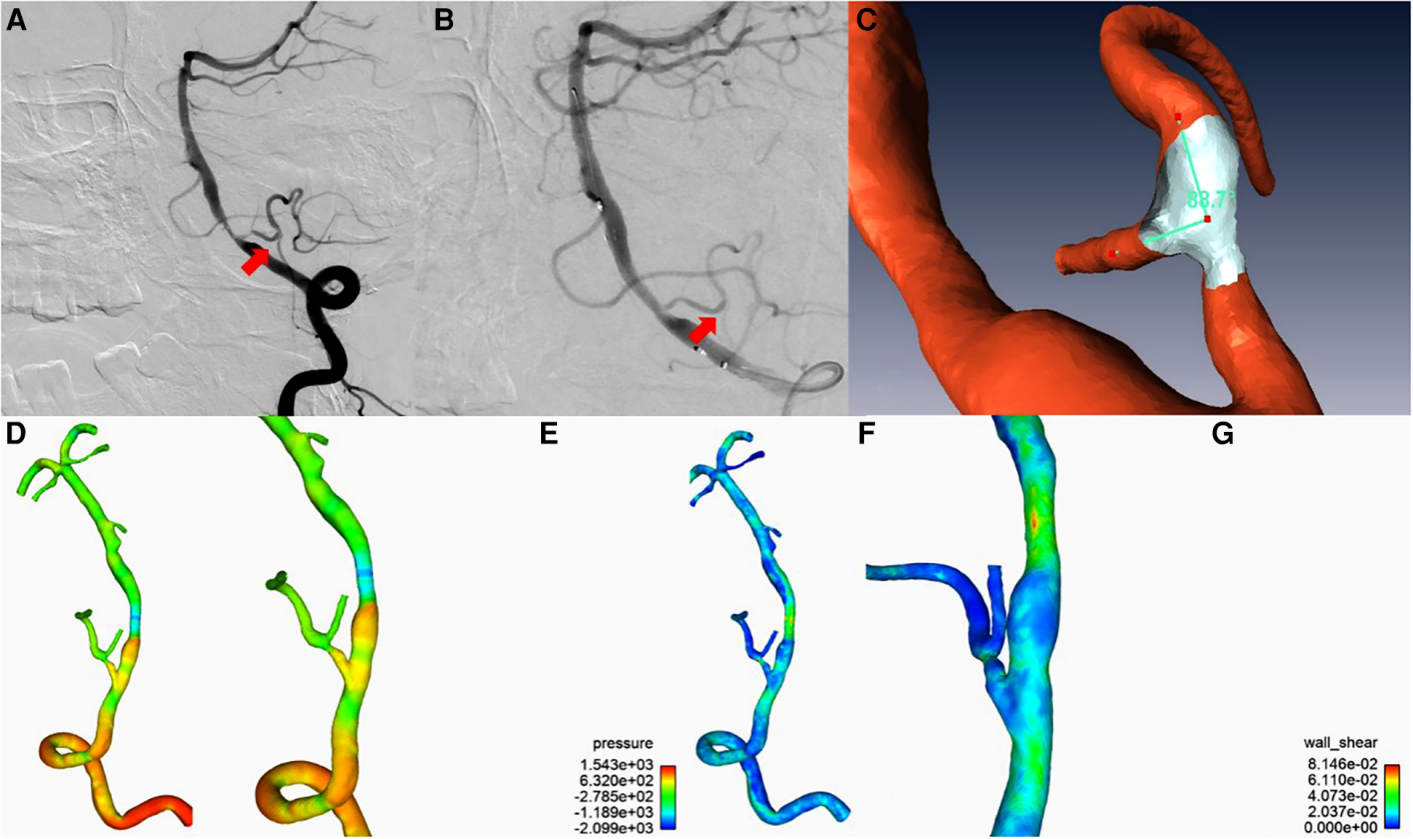
(**A**) A patent graft vessel after the bypass; (**B**) After stenting, the blood supply of the bypass vessel decreased and worsened the imaging quality; (**C**) ANSYS software reconstruction of the intravascular angular relationship between the OA of the bypass vessel and the PCIA of the recipient vessel; (**D,E**) ANSYS software analysis of the bypass vessel and the distal vessels with stable pressure and unobstructed and uniform blood flow; (**F,G**) Angular relationship between the bypass and distal vessels analyzed by ANSYS software, with a low angular force and distal occlusion rate.

Postoperatively, we employed ANSYS software to assess the morphology of the bypass vessels and to calculate the flow pressure and shear force (Figure 3). The results showed stable pressure and unobstructed and uniform flow in the bypass vessels. Meanwhile, the closing angle forces of the bypass and the distal vessels were small, suggesting a low distal occlusion rate of the vessel.

Our patients were followed up for 12–24 months postoperatively, with a mean of 18 months. The three surviving patients had a good prognosis (mRS score of 1) at one year postoperatively, all of whom showed patency of the bridge-vessel anastomosis on repeat CTA or DSA examinations during the follow-up period.

## Discussion

With the technical advances in endovascular treatment, stenting has been increasingly used in severe stenosis or occlusion of the vertebral basilar artery. However, still many risks have been a challenge to this intervention, such as vasospasm, vascular rupture and bleeding, thrombus dislodgement, hyperperfusion syndrome, thrombosis, occlusion of the penetrating artery, and other complications (1). In patients with vertebral artery combined with posterior inferior cerebellar artery stenosis, the plaque stenosis at the beginning of the PICA increases the likelihood of occlusion of the PICA artery during vertebral artery stenting and of the development of lateral medullary syndrome, which is life-threatening in severe cases (2).

Cerebrovascular bypass surgery is a common treatment for intracranial ischemic cerebrovascular disease and complex aneurysms, and although endovascular treatment techniques have been developed in recent years, vascular bypass surgery is still an indispensable treatment (3, 4). The incomplete embolization rate of vertebral artery aneurysms involving the posterior inferior cerebellar artery was reported to reach 72.7%, with a recurrence rate of 14.7% when only endovascular treatment was performed (5, 6). Notably, the chance of serious complications was higher when vertebral and posterior inferior cerebellar artery stenosis was combined. Therefore, in this study, OA-PICA-protected bypass grafting was used to treat cases of stenosis at this site, with a good prognosis (mRS score of 1) rate of 3/3, which is consistent with previous reports in the literature (7). Due to the small number of cases in this operation plan, we will further accumulate cases for in-depth analysis in the future. Hence, OA-PICA-protected bypass grafting is a safe and effective method for treating patients with vertebral artery stenosis involving the posterior inferior cerebellar artery.

Since the first OA-PICA bypass completed by Ausman (8), a variety of bypass types such as STA-SCA have been successively carried out. The main current posterior circulation revascularization procedures applied are as follows:

1. Occipital artery-posterior inferior cerebellar artery anastomosis (OA-PICA bypass), which adopts a distal lateral approach and can fully reveal the VA and PICA. This method provides many options for anastomosis as the length of the occipital artery is sufficient to achieve tension-free anastomosis, with little damage to the surrounding structures (9, 10). At present, this revascularization technique is mainly used to treat PICA artery stenosis occlusive disease or PICA segment aneurysm.However, the proximal end of the occipital artery travels deep in the muscle, which increases the anatomical complexity and requires sharp freeing of the occipital artery under a microscope to prevent spasm occurrence;
2. Lateral anastomosis of the bilateral posterior inferior cerebellar arteries (PICA-PICA bypass), which uses a posterior median approach without grafting vessels. However, its disadvantage is that the anastomosis process requires simultaneous blocking of the bilateral PICAs, combined with a long anastomosis time with an increased risk of ischemic events. Thus, a thrombus formed at the anastomosis, would affect the bilateral PICAs with serious consequences (11);
3. Posterior inferior cerebellar artery and vertebral artery end-lateral anastomosis (PICA-RA-VA bypass), which uses a distal lateral approach and requires preoperative grafting of the radial artery (RA) or saphenous vein (GSV). It is not commonly used clinically because of the deep location of the vessel, the difficulty of the anastomosis, and the tendency of damage to the peripheral nerves (12).

In this study, all three patients underwent end-to-side anastomosis of the occipital artery and the posterior inferior cerebellar artery, followed by stenting of the vertebral artery. Intraoperative angiography was utilized to assess the condition of the bypass vessels. Patency of the anastomosed artery was observed in all three cases. Next, ANSYS software was applied to evaluate the vascular pressure and shear force, which was also employed to estimate the possibility of long-term occlusion and patency of the bridge vessels and to provide a basis for postoperative patient follow-up examinations.

Although posterior circulation revascularization is a more difficult procedure than anterior circulation revascularization, the application of OA-PICA-protected bypass grafting in patients with vertebral artery combined with posterior inferior cerebellar artery stenosis is safe and effective. This approach can considerably reduce the incidence of infarction and mortality due to occlusion during endovascular treatment.

## Data Availability

The original contributions presented in the study are included in the article/Supplementary Material, further inquiries can be directed to the corresponding author/s.
